# Issues on microbial soil remediation: a case of Cd detoxification by *Bacillus* strains for alleviating heavy metal stress in crop plants

**DOI:** 10.3389/fmicb.2025.1665354

**Published:** 2025-09-19

**Authors:** Yini Shi, Xianyang Feng, Zhongke Sun, Boyuan Zhang, Chengwei Li

**Affiliations:** ^1^School of Biological Engineering, Henan University of Technology, Zhengzhou, China; ^2^College of Life Sciences, Zhengzhou University, Zhengzhou, China

**Keywords:** *Bacillus* spp., molecular identification, microbial biosafety, cell number, soil remediation

## Highlights

- Species identification based only on 16S rRNA gene alignment is inadequate for discrimination of isolates within the genus *Bacillus*.
- Biosafety should be evaluated before inoculation of any uncharacterized microbes, especially *B. cereus*.
- Details for cell amount and delivery method are necessary.
- Realistic concentrations of pollutants should be carefully checked and correctly reported.

## Introduction

Microbial remediation is an environmental restoration technique that uses microorganisms to break down or remove pollutants from soil, water, and other environments. This process leverages the natural metabolic capabilities of microbes to degrade contaminants into less harmful or non-toxic substances or to immobilize contaminants and decrease the bioavailability. It is a sustainable and cost-effective approach for cleaning up polluted sites. Among different contaminants, heavy metals such as cadmium (Cd), lead (Pb), arsenic (As), mercury (Hg), and chromium (Cr) are persistent environmental pollutants that pose significant risks to ecosystems and human health. Microbes can interact with these metals through various mechanisms to mitigate their harmful effects by reducing the toxicity, mobility, or concentration of heavy metals in contaminated soils (Nie et al., 2023).

Considering the high mobility, Cd poses a significant health risk when absorbed by plants. The absorption occurs through roots and leaves, leading to Cd accumulation in edible parts of plants (Lin et al., 2024). The accumulation of Cd in edible parts in plants, especially staple food crops like rice and wheat, attracted extensive concerns. Let alone widely recognized direct hazards to humans and animals, a higher concentration of Cd disrupts plant growth and metabolism. In plants, Cd initiates redox actions through the production of free radicals, alters mineral uptake by disturbing water potential, causes chlorosis and mineral deficiencies, and inhibits nitrate reductase activity and ammonia assimilation (Khanna et al., 2022). To cope with Cd stress, plants employ many strategies to limit Cd influx from the soil, including rhizosphere microbial fixation, altered root cell metabolism, membrane efflux, specific transport, chelation, and detoxification, facilitated by various metalloproteins. Considering the vital role of crops in food supply and human health, mainly three approaches were developed for lowering Cd accumulation and improving Cd tolerance in crops, including breeding of resistant cultivars, applying chemical materials, and microbial chelation and detoxification (Zhang et al., 2024).

A recent publication demonstrated effective detoxification of Cd in wheat (*Triticum aestivum*) through the use of an endophytic *Bacillus thuringiensis* and *Salix alba* root powder (Shahzad et al., 2025). However, we found many issues that are critical for the reliability and quality of scientifically sound studies on microbial remediation. Further literature surveys indicated that some of these issues are widely ignored. For simplicity, we discussed these issues with Cd detoxification by *Bacillus* strains as an example. At first, we address the classification of *Bacillus* isolates that were widely identified and named inaccurately. Secondly, we consider the frequently ignored risk after introducing new isolates, especially endophytes, into a different habitat. Thirdly, the amount of exogenous microbial inoculant and delivery method need to be described in detail for repeatability and comparable effect. At last, we highlight the necessity to carefully determine and correctly report the real concentrations of Cd in different settings.

## Cd-tolerant *Bacillus* spp.

With the increase of polluted soils, microorganisms are considered a green and effective means for Cd-contaminated soil remediation. Of them, Cd-tolerant bacteria are microorganisms that can survive and thrive in environments with high concentrations of Cd (Bravo and Braissant, 2022). Some examples of Cd-tolerant bacteria include *Bacillus* spp. and *Rhizobium* spp. that are often found in contaminated soils and capable of Cd sequestration. These bacteria have developed mechanisms to tolerate or detoxify Cd, making them valuable for bioremediation and environmental cleanup. Cd-tolerant bacteria can be used to clean up Cd-contaminated soil and water by immobilizing or transforming the metal into less toxic forms (Ma et al., 2023). They can also be inoculated for alleviating Cd stress in plants and paired with plants to improve the efficiency of phytoremediation in contaminated environments.

To be more focused, we only discuss diverse *Bacillus* strains that were reported for alleviating Cd stress and its accumulation in food crops. Due to their wide presence and versatile effects, strains belonging to *Bacillus* spp. were frequently isolated and studied for soil remediation (Supplementary Table 1). For example, growth promotion and Cd reduction in rice, wheat, and maize were reported after treatment by *B. cereus, B. subtilis*, and *B. pumilus*, respectively (Jabeen et al., 2022; Maslennikova et al., 2023; Shafiq et al., 2022). In addition, beneficial effects of *B. megaterium* on peanuts and *B. cereus* on bananas were also demonstrated under Cd stress (Yao et al., 2021; Zhang et al., 2023). The reasons for these effects are versatile, such as biofilm formation, cell wall binding, extracellular precipitation, intracellular sequestration, and enzyme detoxification (Fu et al., 2024). Briefly, biofilms can protect bacterial communities from Cd toxicity by creating a barrier and enhancing collective resistance. The bacterial cell wall can adsorb Cd ions, preventing them from entering the cell. Some bacteria can precipitate Cd as insoluble compounds (e.g., CdS or CdCO_3_) outside the cell, reducing its bioavailability. Some bacteria produce metal-binding proteins, peptides, and enzymes that bind Cd ions, rendering them less toxic. Some bacteria also have efflux pumps that can actively transport Cd ions out of the cell, reducing intracellular concentrations and preventing toxicity.

## Phylogenetic classification of Cd-resistant *Bacillus* strains

The genus *Bacillus* belongs to the phylum *Firmicutes* and includes a diverse group of gram-positive, rod-shaped, spore-forming bacteria. Based on an updated list, *Bacillus* has more than 500 child taxa with a validly published name under the International Code of Nomenclature of Prokaryotes (ICNP), and its species are classified into several phylogenetic groups (https://lpsn.dsmz.de/genus/bacillus). Two common groups include *the Bacillus subtilis* group (such as *B. subtilis, B. amyloliquefaciens*, and *B. licheniformis*) and *the B. cereus* group (such as *B. cereus, B. thuringiensis, B. anthracis*, and *B. mycoides*). The phylogenetic classification of *Bacillus* species is normally based on genetic, phenotypic, and biochemical characteristics. For genetic characterization, several phylogenetic tools had been developed for bacterial classification. Among them, 16S rRNA gene alignment is one of the most widely applied methods; however, its resolution is limited for the genus *Bacillus*. Due to more markers, Multi-Locus Sequence Typing (MLST) provides higher resolution for closely related species. Although Whole-Genome Sequencing (WGS) offers comprehensive insights into evolutionary relationships, the classification of *Bacillus* remains challenging due to its complex and ever-evolving taxonomic framework, despite its prevalence in nature (Xu and Kovács, 2024).

A recent work identified an isolate as *B. thuringiensis* that showed 97% similarity with other *Bacillus* strains based on 16S rRNA sequence (Shahzad et al., 2025). However, we see its close evolutionary relationship with *B. mycodies* on the neighbor-joining tree. To verify our speculation, the deposited sequence (Accession: MW979616, described as *B. thuringiensis* strain endophytic 04 16S ribosomal RNA gene, partial sequence, 700 bp) on the NCBI website was blasted using the 16S ribosomal RNA (Bacteria and Archaea type strains) database. It is clear that the isolate cannot be credibly classified due to more than 99% identity to a large number of different species (the top ten hits can be found in Supplementary Table 2). In fact, more than twenty two different species have identities higher than 97% to the isolate.

For microbiologists, phylogenetic analysis helps species delineation and novel strain identification, making the characterization and phylogeny of the *Bacillus* genus mutually informative and complementary. To check whether inaccurate classification of *Bacillus* is an ignored problem in previous studies, we performed a narrative literature survey by searching PubMed with keywords “bacillus AND Cd” in the title and published between the years 2022 and 2024. Of twenty research articles using *Bacillus* spp. for soil Cd remediation, ten reports identified *Bacillus* isolates at the species level based only on the 16S rRNA gene, and all names are inconsistent with their top hits after BLAST. These data mean imprecise designation of new *Bacillus* isolates is common in environmental studies (Supplementary Table 3).

## Safety and risk of cd-tolerant *Bacillus* strains

**Figure 1:**
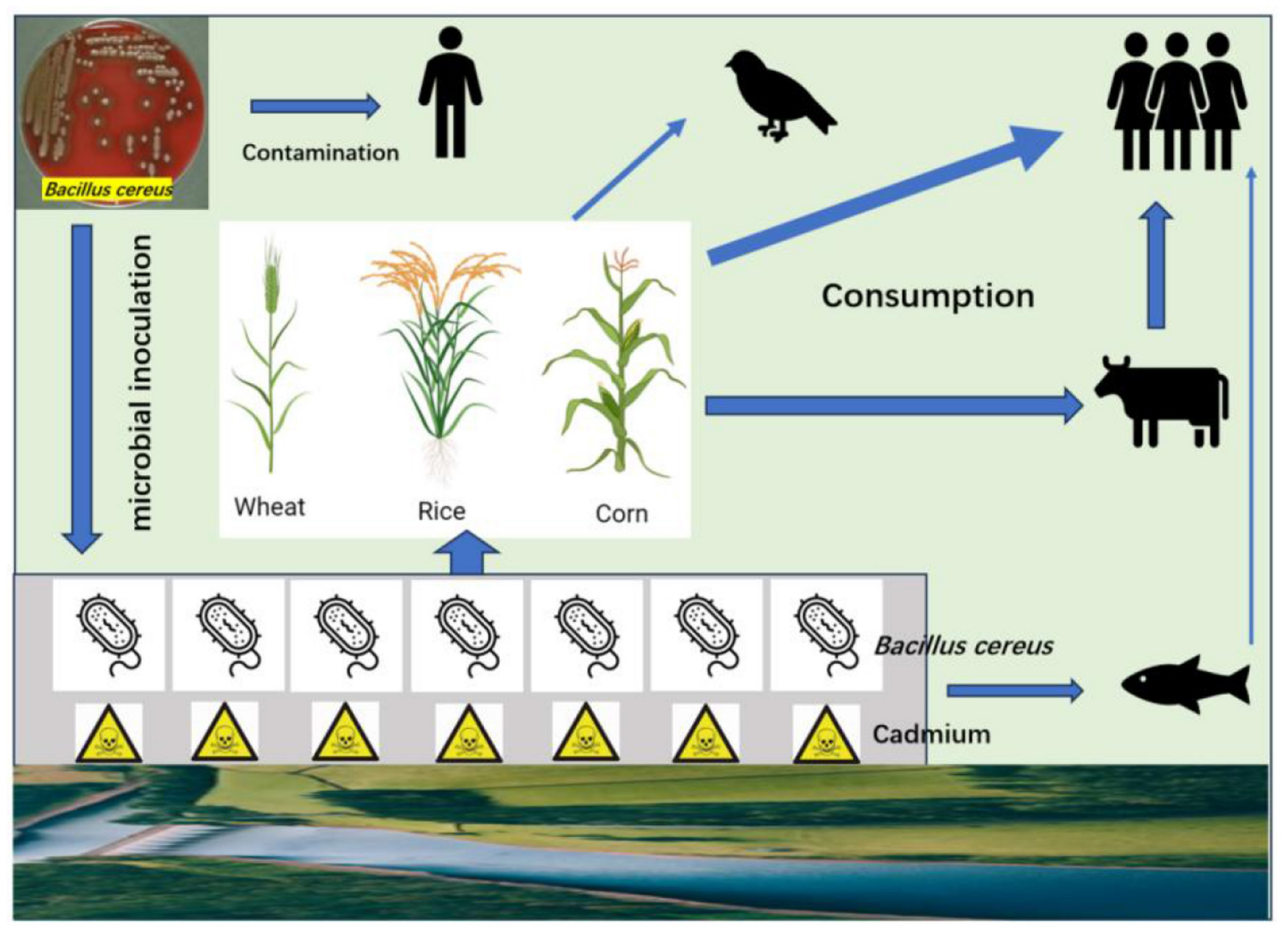
Illustration of *B. cereus* contamination and transmission in the environment. Humans may be infected during the manipulation and production of *B. cereus*. After released into soil as microbial inoculant, *B. cereus* can replicate in soil and absorb Cd. Some strains of *B. cereus* may enter or adhere plant tissues, becoming endophytes or associated microbes. The grains of staple crops are consumed by human and birds, while the stems and leaves can be consumed by animals. *B. cereus* can also be transmitted by water into marine products. All these processes pose a threat to the environmental health.

Cd-tolerant bacteria are a promising tool for addressing Cd pollution, but their use requires careful consideration of environmental and safety factors due to potential unintended consequences. The evaluation of bacterial safety is a critical step in both environmental and agricultural applications, particularly when these ecosystems are closely associated with foods and humans. This assessment ensures that bacterial strains do not pose risks to human health, animal welfare, and ecological balance. Unfortunately, most reports did not consider the safety levels of isolated strains before application in the environment. The endophytic strain isolated from *Salix alba*, identified as *B. thuringiensis*, is closely associated with *B. cereus* (Shahzad et al., 2025). The *B*. *cereus* group encompasses a wide array of pathogenic strains, causing food spoilage and human disease, as well as invertebrate death. As illustrated, releasing a large amount of *B. cereus* may pose a severe threat to environmental health (Figure 1).

In fact, strains belonging to *B*. *cereus* are denied for registration, and the Ministry of Agriculture and Rural Affairs prohibits the development of agents containing *B*. *cereus* for biofertilization or soil remediation in China due to their hemolytic potential (http://www.moa.gov.cn). As one of the important opportunistic foodborne pathogens, *B. cereus* group bacteria with multiple antibiotic resistance genes were detected in a variety of foods, especially cereal flour and wheat/rice noodles (Zheng et al., 2024). In addition, the majority of strains belonging to the *B. cereus* group possess the diarrheal virulence genes *nheABC, hblACD*, and *cytK*. Therefore, it is critical to implement a structured evaluation framework for bacterial safety assessment. Evaluating bacterial safety requires a multi-faceted approach combining genomic analysis, phenotypic testing, ecological modeling, and adherence to regulatory standards. Minimally, the framework needs to evaluate the pathogenicity to humans and animals and the environmental impact. For applications like bioremediation, non-pathogenic strains with minimal ecological disruption potential are preferred. Continuous monitoring and adaptive risk management are essential to address emerging challenges.

### Human and animal pathogenicity

For pathogenicity assessment, determination of whether the bacterium is classified as a pathogen to humans and animals using databases like the NCBI Pathogen Detection Isolates Browser, Pathogenicity Island Database (PAIDB), or Virulence Factors of Pathogenic Bacteria Database (VFDB), is easy and can be a prerequisite before its application in the environment. Based on 16S rRNA alignment, the strain isolated by Shahzad et al. has the highest identity to *B. cereus* and *B. thuringiensis* (Shahzad et al., 2025). In the *B. cereus* group, more than seven thousand isolates were recorded in the pathogen database (https://www.ncbi.nlm.nih.gov/pathogens/organisms/). In the PAIDB, a few representatives of them also have pathogenicity island-like regions (http://www.paidb.re.kr/browse_genomes.php?m=g). The virulence genes (e.g., toxins, adhesion factors) of different *B. cereus* strains were also commonly present in the VFDB. In fact, some *B. cereus* strains cause localized wound and eye infections as well as systemic diseases and are commonly recognized as food poisoning agents. Certain *B. thuringiensis* strains occasionally cause infections in immunocompromised individuals (Ehling-Schulz et al., 2019). In our opinion, it is unacceptable to mitigate a hazard by introducing another danger, which might be infectious and more harmful.

### Environmental impacts

For environmental impact assessment, the potential to outcompete native microbial communities or alter biogeochemical cycles and the persistence should be assessed, e.g., the determination of whether the bacterium (spore-forming *Bacillus* spp.) disrupts the homeostasis of the environment. In one report, *B. thuringiensis* could significantly increase the soil bioavailable Cd content and effectively compensate for alkali-hydro nitrogen losses and microbial inhibition caused by Cd. Furthermore, bacterial inoculation improved bacterial community structure and the relative abundance of Cd-resistant bacteria and changed soil enzyme activity (Chen et al., 2024). In another report, adverse effects of *B. thuringiensis* subsp. *israelensis* (Bti), a widely considered environmentally friendly strain, have been observed in non-biting midges of the family *Chironomidae*. A notable decrease (47% and 41%, respectively) in interspecific diversity of *Chironominae* was detected after Bti treatment in two out of three microhabitats (Stoll et al., 2025). Different from previous reductions, it was also demonstrated that Cd-tolerant endophytic *B. cereus* T4 isolated from rice seeds greatly increased the Cd content of rice roots and above-ground sections in hydroponic pot trials by 158.19% and 140.79%, respectively (Li et al., 2023). These outcomes highlight the impact variability of *Bacillus* spp. and emphasize the necessity for comprehensive risk assessments that encompass diversity at various taxonomic levels and environmental variation at different spatial scales.

## Cell number and delivery of cd-tolerant *Bacillus* strains

Microbial remediation offers advantages like environmental friendliness, cost-effectiveness, and simple operation. However, the efficacy of this remediation process relies on obtaining dominant strains and competitive colonization of niches (Yan et al., 2024). Although the exact number of bacterial cells required for soil remediation depends on several factors, including the type of contaminant, the specific bacterial strain being used, soil conditions (e.g., pH, moisture, organic matter), and the extent of contamination, general guidelines and considerations can help determine the appropriate inoculum size. Typically, soil inoculant requires a density of 10^6^ to 10^8^ CFU/g of soil for efficient competition and survival (Papin et al., 2024). For heavily contaminated soils, higher cell densities (e.g., 10^8^ to 10^9^ CFU/g) may be necessary. Anyhow, to optimize the inoculum size, small-scale trials by monitoring bacterial survival and contaminant degradation after inoculation may be necessary. In some cases, using endophytes or adding nutrients (like *Salix alba* root powder) may enhance the activity and reduce the need for large inoculum sizes. However, only about 10^3^ bacterial cells may have little real effect, especially when the soil is heavily contaminated and the concentration of Cd is far above the minimum inhibitory concentration (Shahzad et al., 2025).

On the other side, the delivery approaches are also important from a practical point. Due to the rapid decline of the population and activity after direct soil inoculation, most microbial agents face a dilemma on stability and effectiveness (Liu et al., 2023). To solve this problem, two approaches are preferred. One is coating microbes on the seed of plants. For example, to detoxify and promote chickpea growth in Cd-contaminated soils, about 10^8^ *Pseudomonas fluorescens* PGPR-7 cells were delivered per seed using 1% guar gum powder as an adhesive (Syed et al., 2023). The other is immobilization with carriers. Environmentally compatible carriers, like biochar or sodium alginate, might be good choices. It is reported that biochar successfully facilitated the growth of *B. megaterium* and Cd immobilization (Qi et al., 2023). Let alone decrease Cd, the *B. cereus*/biochar composite significantly increased the soil pH by about 1.5 units and the activities of catalase, urease, and invertase enzymes (Mei et al., 2022). Also, we showed that sodium alginate can enhance the growth and enzyme activity of applied *Bacillus sp*. in the soil (Sun et al., 2024).

## Cd concentration and its determination

**Figure 2:**
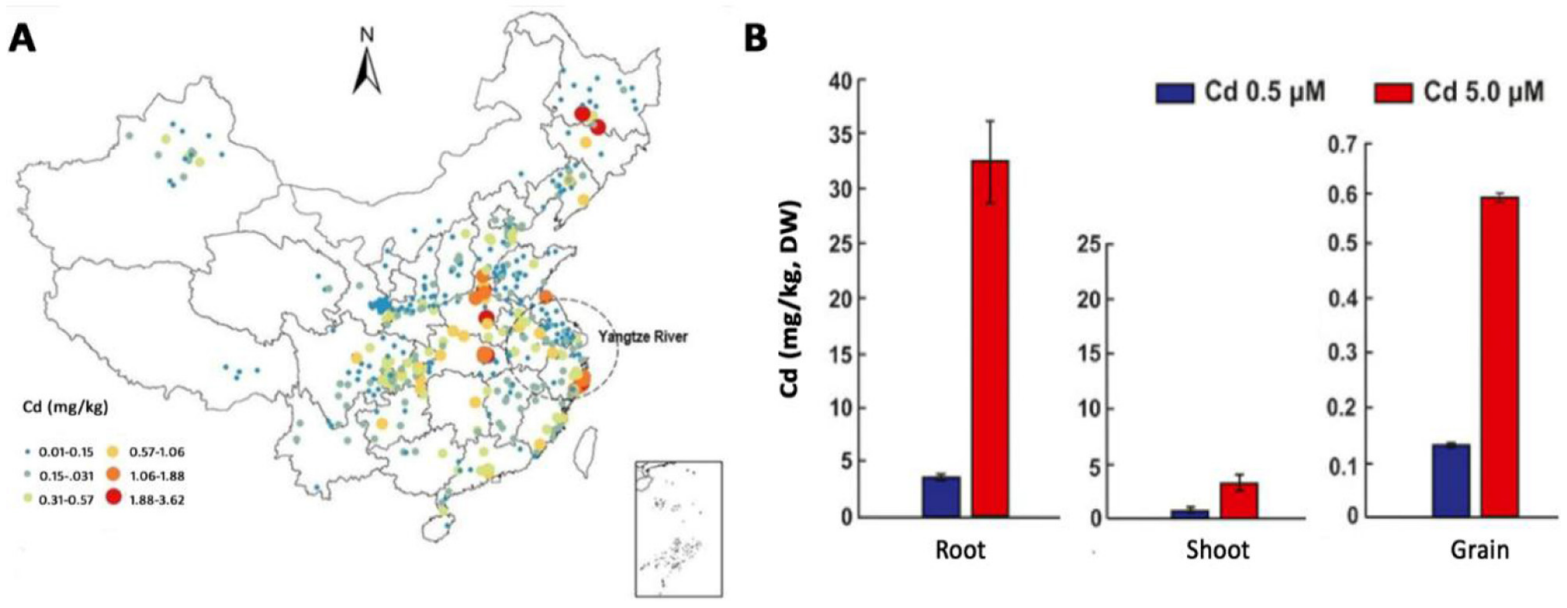
The concentration of Cd in soils and wheat tissues. **(A)**, Cd concentration levels in agricultural soils across China. **(B)**, Cd in the root, shoot, and grain of wheat when grown in soils added 0.5 or 5.0 μM Cd. The figure was adapted from two previous articles (Cheng et al., 2023; Sabella et al., 2022).

The concentration of Cd in arable soil is basic information for the evaluation of its toxicity. According to a comprehensive report that summarized four hundred and eighty six studies of Cd concentrations in arable soils, the average Cd concentration was 0.27 mg/kg in China (Zhang et al., 2015). Further analysis suggests the majority of arable soils (45.16%) have Cd with concentrations between 0.097 and 0.3 mg/kg, although more than 150 mg/kg is available in a certain mining area. A more recent study retrieved the concentration data of Cd from six hundred and two sampling sites, revealing that the average Cd concentration was 0.29 mg/kg, which is very close to China's national quality standard of 0.3 mg/kg (standard code: GB15618-2018) for agricultural land (Cheng et al., 2023). Of these sampling sites, 69.1% exceed the national average background concentration of 0.097 mg/kg for Cd by 2.99 times. All these surveys indicated that the contamination of Cd in Chinese agricultural soils is quite prevalent (Figure 2A). The widespread contamination of Cd in agricultural soils inevitably resulted in a considerable proportion of grains having Cd concentrations exceeding the Chinese food limit, raising widespread concern regarding food safety (Wang et al., 2019).

Strikingly, the solution of CdSO_4_ was prepared in two different concentrations (20 and 40 mg/mL) in the recent publication, and about 100 mL of these solutions were added to 1 kg of potted soil (Shahzad et al., 2025). These stand for an average of 2,000 and 4,000 mg/kg CdSO_4_ that are roughly 10,000-fold higher than the concentration in most soils. From our experience, many crops cannot grow under such high concentrations of Cd. For example, in a hydroponic model, 100 μM Cd (~22.85 mg/L) hampered the overall plant growth and development, resulting in noticeable toxicity to rice seedlings, including plant dwarfing, leaf withering, and chlorosis (Gu et al., 2023). When Cd concentration was 0.4 mg/kg in soil, the growth parameters of chickpea were severely influenced, e.g., germination rate was less than 60%, plant length was less than 1/2, and dry weight was less than 1/3 (Syed et al., 2023). Generally, wheat is sensitive to Cd at soil concentrations as low as 0.2–5 mg/kg. Concentrations above 5 mg/kg can cause significant toxicity, reducing growth and yield. As reported, the wheat seed germination rate decreased to less than 60% by 1 mM Cd, and the growth of the endophytic plant growth-promoting bacterium *B. subtilis* 10-4 was completely inhibited under this Cd concentration (Maslennikova et al., 2023).

The concentrations of Cd in plant tissues are different (Figure 2B). According to a review, the average concentration of Cd in wheat grains ranged between 0.0080 mg/kg and 2.0 mg/kg (dry weight), and 83% of these wheat samples have lower Cd concentrations than the national food safety standard of 0.1 mg/kg (Gao et al., 2022). In contrast, under a concentration of 5 μM that is nontoxic for roots but agronomically relevant, Cd concentrations can reach about 30, 5, and 0.6 mg/kg in the root, shoot, and grain, respectively (Sabella et al., 2022). In contrast, 0.17 mg/g (170 mg/kg) Cd was reported in wheat plants grown in soils without contamination of Cd, and as high as 5.99 mg/g (5,990 mg/kg) when soils were contaminated with 40 mg/kg Cd (Shahzad et al., 2025). This means there are more than 100 folds of Cd in wheat than in soils. Thinking wheat is not a Cd hyper accumulator, such a high Cd concentration seems impossible.

Disappointingly, many studies did not provide details on how pollutants were determined. To determine the content of Cd in plants, we believe the sampling and processing methods should be widely accepted by the scientific community. For the extraction of metals, samples should be treated by the most widely used methods. For example, to precisely determine Cd content, finely ground plant tissues (to be representative, 0.1 g is required for sampling) can be dried and digested in a solution containing trace-metal-grade concentrated HNO_3_ and 30% (v/v) H_2_O_2_ in a microwave digestion system. Detection should be conducted with accredited equipment, like atomic fluorescence spectrophotometry (AFS) and inductively coupled plasma atomic emission spectrometry (ICP-AES).

## References

[B1] Bravo D. Braissant O. (2022). Cadmium-tolerant bacteria: current trends and applications in agriculture. Lett. Appl. Microbiol. 74, 311–333. 10.1111/lam.13594 34714944 PMC9299123

[B2] Chen Y. Li S. Chen X. Li Y. Yan C. Wang C.. (2024). Enhanced Cd activation by *Coprinus comatus* endophyte *Bacillus thuringiensis* and the molecular mechanism. Environ. Pollut. 342:123052. 10.1016/j.envpol.2023.123052 38040187

[B3] Cheng Y. Ma J. Li S. Tang Q. Shi W. Liang Y.. (2023). Dietary cadmium health risk assessment for the Chinese population. Environ. Sci. Pollut. Res. Int. 30, 82421–82436. 10.1007/s11356-023-28199-0 37326726

[B4] Ehling-Schulz M. Lereclus D. Koehler T. M. (2019). The *Bacillus cereus* group: *Bacillus* species with pathogenic potential. Microbiol. Spectr. 7:1128. 10.1128/microbiolspec.GPP3-0032-2018 31111815 PMC6530592

[B5] Fu S. Iqbal B. Li G. Alabbosh K. F. Khan K. A. Zhao X.. (2024). The role of microbial partners in heavy metal metabolism in plants: a review. Plant Cell Rep. 43:111. 10.1007/s00299-024-03194-y38568247

[B6] Gao Y. Duan Z. Zhang L. Sun D. Li X. (2022). The status and research progress of Cadmium pollution in rice- (*Oryza sativa* L.) and wheat- (*Triticum aestivum* L.) cropping systems in China: a critical review. Toxics 10:794. 10.3390/toxics10120794 36548627 PMC9783001

[B7] Gu T. Lu Y. Li F. Zeng W. Shen L. Yu R.. (2023). Microbial extracellular polymeric substances alleviate cadmium toxicity in rice (*Oryza sativa* L.) by regulating cadmium uptake, subcellular distribution and triggering the expression of stress-related genes. Ecotoxicol. Environ. Saf. 257, 114958. 10.1016/j.ecoenv.2023.114958 37116453

[B8] Jabeen Z. Irshad F. Habib A. Hussain N. Sajjad M. Mumtaz S.. (2022). Alleviation of cadmium stress in rice by inoculation of *Bacillus cereus*. Peer J. 10:e13131. 10.7717/peerj.13131 35529485 PMC9070326

[B9] Khanna K. Kohli S. K. Ohri P. Bhardwaj R. Ahmad P. (2022). Agroecotoxicological aspect of Cd in soil-plant system: uptake, translocation and amelioration strategies. Environ. Sci. Pollut. Res. Int. 29, 30908–30934. 10.1007/s11356-021-18232-5 35094262

[B10] Li P. Xiong Z. Tian Y. Zheng Z. Liu Z. Hu R.. (2023). Community-based mechanisms underlying the root cadmium uptake regulated by Cd-tolerant strains in rice (*Oryza sativa. L)*. Frontiers in plant science 14, 1196130. 10.3389/fpls.2023.1196130 37636120 PMC10450764

[B11] Lin L. Wu X. Deng X. Lin Z. Liu C. Zhang J.. (2024). Mechanisms of low cadmium accumulation in crops: a comprehensive overview from rhizosphere soil to edible parts. Environ. Res. 245:118054. 10.1016/j.envres.2023.118054 38157968

[B12] Liu Y. Yue Z. Sun Z. Li C. (2023). Harnessing native *Bacillus* spp. for sustainable wheat production. Appl. Environ. Microbiol. 89:e0124722. 10.1128/aem.01247-22 36695599 PMC9973024

[B13] Ma B. Song W. Zhang X. Chen M. Li J. Yang X.. (2023). Potential application of novel cadmium-tolerant bacteria in bioremediation of Cd-contaminated soil. Ecotoxicol. Environ. Saf. 255:114766. 10.1016/j.ecoenv.2023.114766 36924559

[B14] Maslennikova D. Koryakov I. Yuldashev R. Avtushenko I. Yakupova A. Lastochkina O. (2023). Endophytic plant growth-promoting bacterium *Bacillus subtilis* reduces the toxic effect of cadmium on wheat plants. Microorganisms 11:1653. 10.3390/microorganisms11071653 37512826 PMC10386265

[B15] Mei C. Wang H. Cai K. Xiao R. Xu M. Li Z.. (2022). Characterization of soil microbial community activity and structure for reducing available Cd by rice straw biochar and Bacillus cereus RC-1. Sci. Total Environ. 839:156202. 10.1016/j.scitotenv.2022.156202 35623534

[B16] Nie M. Wu C. Tang Y. Shi G. Wang X. Hu C.. (2023). Selenium and *Bacillus proteolyticus* SES synergistically enhanced ryegrass to remediate Cu-Cd-Cr contaminated soil. Environ. Pollut. 323:121272. 10.1016/j.envpol.2023.121272 36780973

[B17] Papin M. Philippot L. Breuil M. C. Bru D. Dreux-Zigha A. Mounier A.. (2024). Survival of a microbial inoculant in soil after recurrent inoculations. Sci. Rep. 14, 4177. 10.1038/s41598-024-54069-x 38378706 PMC10879113

[B18] Qi W. Y. Chen H. Wang Z. Xing S. F. Song C. Yan Z.. (2023). Biochar-immobilized Bacillus megaterium enhances Cd immobilization in soil and promotes Brassica chinensis growth. J. Hazard. Mater. 458:131921. 10.1016/j.jhazmat.2023.131921 37406520

[B19] Sabella E. Aprile A. Tenuzzo B. A. Carata E. Panzarini E. Luvisi A.. (2022). Effects of cadmium on root morpho-physiology of durum wheat. Front. Plant Sci. 13:936020. 10.3389/fpls.2022.936020 35812940 PMC9260267

[B20] Shafiq T. Yasmin H. Shah Z. A. Nosheen A. Ahmad P. Kaushik P.. (2022). Titanium oxide and zinc oxide nanoparticles in combination with cadmium tolerant Bacillus pumilus ameliorates the cadmium toxicity in maize. Antioxidants 11:2156. 10.3390/antiox11112156 36358528 PMC9686562

[B21] Shahzad A. Hameed S. Qin M. Li H. Zafar S. Siddiqui S.. (2025). Cadmium (Cd) detoxification and activation of plant defense enzymes in wheat (*Triticum aestivum*) through the use of endophytic *Bacillus thuringiensis* and *Salix alba* root powder. Environ. Pollut. 364:125147. 10.1016/j.ecoenv.2024.116698 39447632

[B22] Stoll V. S. Röder N. Gerstle V. Manfrin A. Schwenk K. (2025). Effects of Bti on the diversity and community composition of three *Chironomidae* subfamilies across different micro-habitats. Environ. Pollut. 366:125490. 10.1016/j.envpol.2024.125490 39653262

[B23] Sun Z. Feng X. Shi Y. Li C. (2024). Sodium alginate improves phytase stability and enhances soil phosphorous utilization. Biocat. Agricult. Biotechnol. 61:103372. 10.1016/j.bcab.2024.103372

[B24] Syed A. Elgorban A. M. Bahkali A. H. Eswaramoorthy R. Iqbal R. K. Danish S. (2023). Metal-tolerant and siderophore producing *Pseudomonas fluorescence* and *Trichoderma* spp. improved the growth, biochemical features and yield attributes of chickpea by lowering Cd uptake. Sci. Rep. 13:4471. 10.1038/s41598-023-31330-3 36934106 PMC10024765

[B25] Wang P. Chen H. Kopittke P. M. Zhao F. J. (2019). Cadmium contamination in agricultural soils of China and the impact on food safety. Environ. Pollut. 249, 1038–1048. 10.1016/j.envpol.2019.03.063 31146310

[B26] Xu X. Kovács Á. T. (2024). How to identify and quantify the members of the Bacillus genus. Environ. Microbiol. 26:e16593. 10.1111/1462-2920.16593 38383138

[B27] Yan Z. X. Li Y. Peng S. Y. Wei L. Zhang B. Deng X. Y.. (2024). Cadmium biosorption and mechanism investigation using two cadmium-tolerant microorganisms isolated from rhizosphere soil of rice. J. Hazard. Mater. 470:134134. 10.1016/j.jhazmat.2024.134134 38554514

[B28] Yao X. Chen P. Cheng T. Sun K. Megharaj M. He W. (2021). Inoculation of *Bacillus megaterium* strain A14 alleviates cadmium accumulation in peanut: effects and underlying mechanisms. J. Appl. Microbiol. 131, 819–832. 10.1111/jam.14983 33386698

[B29] Zhang L. Hu Y. Chen Y. Qi D. Cai B. Zhao Y.. (2023). Cadmium-tolerant Bacillus cereus 2-7 alleviates the phytotoxicity of cadmium exposure in banana plantlets. Sci. Total Environ. 903:166645. 10.1016/j.scitotenv.2023.166645 37657542

[B30] Zhang Q. H. Chen Y. Q. Li Z. B. Tan X. T. Xin G. R. He C. T. (2024). Defense guard: strategies of plants in the fight against Cadmium stress. Adv. Biotechnol. 2:44. 10.1007/s44307-024-00052-6 39883385 PMC11740865

[B31] Zhang X. Chen D. Zhong T. Zhang X. Cheng M. Li X. (2015). Assessment of cadmium (Cd) concentration in arable soil in China. Environ. Sci. Pollut. Res. Int. 22, 4932–4941. 10.1007/s11356-014-3892-6 25483971

[B32] Zheng Z. Ye L. Xiong W. Hu Q. Chen K. Sun R.. (2024). Prevalence and genomic characterization of the *Bacillus cereus* group strains contamination in food products in Southern China. Sci. Total Environ. 921:170903. 10.1016/j.scitotenv.2024.170903 38354793

