# Supplementary material for: Issues on microbial soil remediation: a case of Cd detoxification by Bacillus strains for alleviating heavy metal stress in crop plants
Source: Front Microbiol. 2025 Sep 19;16:1665354. doi: 10.3389/fmicb.2025.1665354 (PMC12491980; doi:10.3389/fmicb.2025.1665354)
Supplement: Supplementary file 1 [file Supplementary_file_1.docx]

Supplementary information

**Issues on microbial soil remediation: a case of Cd detoxification by *Bacillus* strains for alleviating heavy metal stress in crop plants**

Yini Shi^1^, Xianyang Feng^1^, Zhongke Sun^1*^, Boyuan Zhang^1^, Chengwei Li^1,2, *^

School of Biological Engineering, Henan University of Technology, Zhengzhou, 450001, China

College of Life Sciences, Zhengzhou University, Zhengzhou, 450001, China

* Correspondence authors

Prof. Zhongke Sun, Email: sunzh@daad-alumni.de;

ORCID: <https://orcid.org/0000-0002-9784-9769>

Post address: No.100, Lianhua Road, Zhengzhou City, Henan Province, 450001, CN

Prof. Chengwei Li, Email: [lcw@haut.edu.cn](mailto:lcw@haut.edu.cn);

Post address: No.100, Science Road, Zhengzhou City, Henan Province, 450001, CN

Table S1 Some representative Cd-tolerant *Bacillus* spp. used for alleviating Cd stress in different crop plants

| **Strain** | **Model** | **Plants** | **Results** | **Reference** |
| --- | --- | --- | --- | --- |
| ***B. cereus*** | Pot, hydroponic | rice | 33% increase in fresh and dry root weight, 83% higher leaves water content, 230% in leaves and 276% in roots of SOD | Jabeen et al., 2022 |
| ***B. subtilis* 10-4** | Glass, hydroponic | wheat | Reduced Cd concentration both in the roots (by 2 times) and in the shoots (by 3.1 times) | Maslennikova et al., 2023 |
| ***B. pumilus*** | Pot, soil | maize | Improved dry weight (by 3.3 times) and germination index (by 2.67 times) | Shafiq et al., 2022 |
| ***B. megaterium* A14** | Pot, soil | *peanut* | shoot and root biomass increased ~60% and 58%, Cd decreased 48% in grains, exchangeable Cd decreased from 40 to 26% in soil . | Yao et al., 2021 |
| ***B. cereus* 2-7** | Pot, soil | banana | Cd contents decreased 50.31 % in soil, 45.43 % in roots, 56.42 % in stems and 79.69 % in leaves. | Zhang et al., 2023 |

Table S2 The top ten hits of BLASTn using sequence MW979616 as a query

| \| Species name \| Coverage (%) \| Identity (%) \| Mismatches \| Accession No. \| \| --- \| --- \| --- \| --- \| --- \| \| *B. cereus* \| 97 \| 99.71 \| 1 \| [NR_112630.1](https://www.ncbi.nlm.nih.gov/nucleotide/NR_112630.1?report=genbank&log$=nucltop&blast_rank=1&RID=UD8DG5D9013) \| \| *B. thuringiensis* \| 97 \| 99.71 \| 1 \| [NR_112780.1](https://www.ncbi.nlm.nih.gov/nucleotide/NR_112780.1?report=genbank&log$=nucltop&blast_rank=2&RID=UD8DG5D9013) \| \| *B. pacificus* \| 98 \| 99.57 \| 0 \| NR_157733.1 \| \| *B. mobilis* \| 98 \| 99.57 \| 0 \| NR_157731.1 \| \| *B. wiedmannii* \| 98 \| 99.57 \| 0 \| NR_152692.1 \| \| *B. toyonensis* \| 98 \| 99.57 \| 0 \| NR_121761.1 \| \| *B. mycoides* \| 97 \| 99.56 \| 1 \| [NR_113990.1](https://www.ncbi.nlm.nih.gov/nucleotide/NR_113990.1?report=genbank&log$=nucltop&blast_rank=10&RID=UD8DG5D9013) \| \| *B. paranthracis* \| 98 \| 99.42 \| 1 \| NR_157728.1 \| \| *B. proteolyticus* \| 98 \| 99.42 \| 1 \| NR_157735.1 \| \| *B. paramobilis* \| 98 \| 99.42 \| 1 \| NR_175556.1 \| |
| --- | --- | --- | --- | --- | --- | --- | --- | --- | --- | --- | --- | --- | --- | --- | --- | --- | --- | --- | --- | --- | --- | --- | --- | --- | --- | --- | --- | --- | --- | --- | --- | --- | --- | --- | --- | --- | --- | --- | --- | --- | --- | --- | --- | --- | --- | --- | --- | --- | --- | --- | --- | --- | --- | --- | --- |

Table S3 Some Cd-tolerant *Bacillus* isolates inaccurately identified at the species level in literature

| **Isolate** | **Accession** | **Proposed species name** | **Top blasted name** | **Identity (%)** | **Reference** |
| --- | --- | --- | --- | --- | --- |
| R27 | [MF620076.1](https://blast.ncbi.nlm.nih.gov/Blast.cgi) | *B. siamensis* | *[B. stercoris](https://www.ncbi.nlm.nih.gov/Taxonomy/Browser/wwwtax.cgi?id=2054641" \t "https://blast.ncbi.nlm.nih.gov/lnk<@rid@>" \o "Taxonomy for Bacillus stercoris)* | 99.45 | Liu et al., 2024 |
| 04 | MW979616.1 | *B. thuringiensis* | *B. ceres* | 99.71 | Shahzad et al., 2025 |
| - | [KF859972](https://www.ncbi.nlm.nih.gov/nuccore/KF859972).1 | *B. pumilus* | *[B. aerius](https://www.ncbi.nlm.nih.gov/Taxonomy/Browser/wwwtax.cgi?id=293388" \t "https://blast.ncbi.nlm.nih.gov/lnk<@rid@>" \o "Taxonomy for Bacillus aerius)* | 98.62 | Ali et al., 2024 |
| X30 | KU219949.1 | *B. thuringiensis* | *[B. toyonensis](https://blast.ncbi.nlm.nih.gov/Blast.cgi#alnHdr_659364953)* | 100 | Zhu et al., 2024 |
| VITMSJ3 | MT822866.1 | *B. xiamenensis* | *B. altitudinis* | 98.85 | Wagh et al., 2024 |
| SES | OP090574.1 | *B. proteolyticus* | *B. wiedmannii* | 100 | Nie et al., 2023 |
| HM-311 | MN220629.1 | *B. thuringiensis* | *B. cereus* | 97.91 | Zuo et al., 2022 |
| B12 | MH521166.1 | *B. subtilis* | *B. stercoris* | 100 | Ge et al., 2022 |
| RC-1 | [JF683581](http://www.ncbi.nlm.nih.gov/entrez/query.fcgi?cmd=search&db=nucleotide&doptcmdl=genbank&term=JF683581) | *B. cereus* | *[B. paramycoides](https://blast.ncbi.nlm.nih.gov/Blast.cgi#alnHdr_1441204319)* | 98.81 | Mei et al., 2022 |

References

1. Liu, S., Huang, Y., Zheng, Q., Zhan, M., Hu, Z., Ji, H., Zhu, D., & Zhao, X. (2024). Cd-Resistant plant growth-promoting rhizobacteria *Bacillus siamensis* R27 absorbed Cd and reduced Cd accumulation in lettuce (*Lactuca sativa* L.). *Microorganisms,12*(11):2321. https://doi.org/10.3390/microorganisms12112321
2. Shahzad, A., Hameed, S., Qin, M., Li, H., Zafar, S., Siddiqui, S., Sattar, S., Mahmood, Z., & Mehwish, S. (2025). Cadmium (Cd) detoxification and activation of plant defense enzymes in wheat (T*riticum aestivum*) through the use of endophytic *Bacillus thuringiensis* and *Salix alba* root powder. *Environmental pollution (Barking, Essex: 1987)*, *364*(Pt 1), 125147. <https://doi.org/10.1016/j.envpol.2024.125147>
3. Ali, M.A., Nafees, M., Waseem, M., Alomrani, S.O., Al-Ghanim, K.A., Alshehri, M.A., Zheng, H., Ali, S., & Li, F. (2024). Modulation of Cd carriers by innovative nanocomposite (Ca+Mg) and Cd-resistance microbes (*Bacillus pumilus*): a mechanistic approach to enhance growth and yield of rice (*Oryza sativa* L.). *Frontiers in plant science*, *15*:1387187. https://doi.org/10.3389/fpls.2024.1387187
4. Zhu, X., Beiyuan, J., Ju, W., Qiu, T., Cui, Q., Chen, L., Chao, H., Shen, Y., & Fang, L. (2024). Inoculation with *Bacillus thuringiensis* reduces uptake and translocation of Pb/Cd in soil-wheat system: A life cycle study. *The Science of the total environment*, *945*:174032. https://doi.org/10.1016/j.scitotenv.2024.174032
5. Wagh, M.S., Sivarajan, S., & Osborne, J.W. (2024). Deciphering the enhanced translocation of Pb, Ni and Cd from artificially polluted soil to Chrysopogon zizanioides augmented with *Bacillus xiamenensis* VITMSJ3. *3 Biotech, 14*(7):180. https://doi.org/10.1007/s13205-024-04001-x
6. Zhang, H., Nie, M., Du, X., Chen, S., Liu, H., Wu, C., Tang, Y., Lei, Z., Shi, G., & Zhao, X. (2024). Selenium and *Bacillus proteolyticus* SES increased Cu-Cd-Cr uptake by yegrass: highlighting the significance of key taxa and soil enzyme activity. *Environmental science and pollution research international*, *31*(20):29113-29131. https://doi.org/10.1007/s11356-024-32959-x
7. Qi, W.Y., Chen, H., Wang, Z., Xing, S. F., Song, C., Yan, Z., & Wang, S. G. (2023). Biochar-immobilized *Bacillus megaterium* enhances Cd immobilization in soil and promotes *Brassica chinensis* growth. *Journal of hazardous materials*, *458*, 131921. <https://doi.org/10.1016/j.jhazmat.2023.131921>
8. Zuo, W., Song, B., Shi, Y., Zupanic, A., Guo, S., Huang, H., Jiang, L., & Yu, Y. (2022). Using *Bacillus thuringiensis* HM-311@hydroxyapatite@biochar beads to remediate Pb and Cd contaminated farmland soil. *Chemosphere, 307(Pt)*:135797. https://doi.org/10.1016/j.chemosphere.2022.135797
9. Ge, Y., Ge, Z., Zheng, J., Sheng, X., & He, L. (2022). Biofilm-overproducing *Bacillus subtilis* B12ΔYwcc decreases Cd uptake in Chinese cabbage through increasing Cd- immobilizing related gene abundance and root surface colonization. *Journal of Environmental Science (China), 120*:84-93. https://doi.org/10.1016/j.jes.2022.01.016
10. Mei, C., Wang, H., Cai, K., Xiao, R., Xu, M., Li, Z., Zhang, Z., Cui, J., & Huang, F. (2022). Characterization of soil microbial community activity and structure for reducing available Cd by rice straw biochar and *Bacillus cereus* RC-1. *The Science of the total environment*, *839*:156202. https://doi.org/10.1016/j.scitotenv.2022.156202
